# Preeclampsia risk prediction model for Chinese pregnant women (ChiPERM): research protocol for a randomized stepped-wedge cluster trial

**DOI:** 10.1186/s12884-022-04858-x

**Published:** 2022-06-29

**Authors:** Qiongjie Zhou, Jinghui Xu, Yu Xiong, Xiaotian Li

**Affiliations:** 1 Obstetrics and Gynecology Hospital, Fudan University, Shanghai, 200011 China; 2 Shanghai Key Laboratory of Female Reproductive Endocrine-Related Diseases, Shanghai, China

**Keywords:** Preeclampsia, Placenta growth factor, Risk prediction, Aspirin, Pregnancy, Antenatal care, Stepped-wedge trial, Protocol

## Abstract

**Background:**

Despite international clinical guideline recommendations, implementation of Bayes-theorem based preeclampsia risk prediction model in first trimester among Chinese women is limited. The aim of this study is to examine the effectiveness of this risk predictive strategy in reducing the risk of preeclampsia.

**Methods:**

The study will be a randomized, stepped-wedge controlled trial conducted in eighteen hospitals in China. Stepped implementation of Bayes-theorem based risk prediction model will be delivered to hospitals in a random order to support the introduction of this prediction model of preeclampsia. A staged process will be undertaken to develop the risk prediction strategies, which comprise of: combined risk evaluation by maternal risk factors, medium arterial pressure, uterine artery pulse index and placenta growth factor during 11–13^+6^ gestational weeks, monthly follow up (including blood pressure, newly onset complications, adherence to aspirin). Repeated cross-sectional outcome data will be gathered weekly across all hospitals for the study duration. The primary outcome measures are the incidence of preeclampsia within 42 days postpartum. Data on resources expended during intervention development and implementation will be collected. The incidence of pregnancy related complications will be measured as secondary outcomes.

**Discussion:**

This will be the first randomized controlled trial to evaluate the effectiveness of the Bayes-theorem based preeclampsia risk prediction strategies in first trimester by competing risk model validation. If positive changes in clinical practice are found, this evidence will support health service adoption of this risk prediction model to reduce the risk of preeclampsia among Chinese pregnant women.

**Trial registration:**

Chinese Clinical Trials Registry, No. ChiCTR2100043520 (date registered:21/2/2021).

## Background

Preeclampsia is a common obstetrical complication, affecting 2%-8% of all pregnancies [1, 2]. It accounts for maternal and infant morbidity and mortality worldwide. Since first case report in 1978 [3], prevention of preeclampsia with aspirin has been studied for decades [4]. Its effect is inconsistent in abundant case–control, prospective cohort, or meta-analysis studies [5–12]. In the trial of low-dose Aspirin in the Prevention of Pre-Eclampsia in China (APPEC), the strategy of a dose of 100 mg aspirin daily in preventing preeclamapsia among high-risk women screened with maternal risk factors did not reduce the incidence of preeclampsia, indicating further consideration combining with maternal biophysical parameters [13]. In 2019, the International Federation of Gynecology and Obstetrics (FIGO), has recommended a Bayes theorem-based model, which integrated with maternal risk factors, mean arterial pressure (MAP), uterine artery PI (UTPI) and serum placenta growth factor (PlGF) [14]. It has provided a new era for the preeclampsia risk prediction.

Despite clear recommendations in clinical guidelines of preeclampsia risk prediction, most evidence are based in Caucasians or African Americans [5, 9–12, 15]. In the randomized controlled study-the Aspirin for Evidence-Based Preeclampsia Prevention trial based in European population, the incidence of preterm preeclampsia has been reduced by 62% for women at high risk of preeclampsia, when they take aspirin at a daily dosage of 150 mg initiated before 16 gestational week [9]. However, studies addressing the effectiveness of this Bayes theorem-based model among Chinese pregnant women are limited. There was a prospective, non-intervention, multiple centered trial conducted among 10,935 Asian women, but only approximately 700 women from Jiangsu and Yunnan province in mainland China have been recruited [16]. Thus, the effectiveness of this model across China have not been well investigated. Considering varied incidence rates among women of different race and genetic susceptibility, it is of importance to verify the performance of this Fetal Medicine Foundation (FMF) Bayes-theorem based method for Chinese women.

 Accordingly, we have initiated this randomized stepped-wedge cluster trial of Preeclampsia risk prediction model for Chinese pregnant women (ChiPERM). The aim of this study is to examine the effectiveness of this Bayes-theorem based risk prediction model in first trimester for reducing the risk of preeclampsia among Chinese pregnant women.

## Methods/design

### Study design and setting

**Fig. 1:**
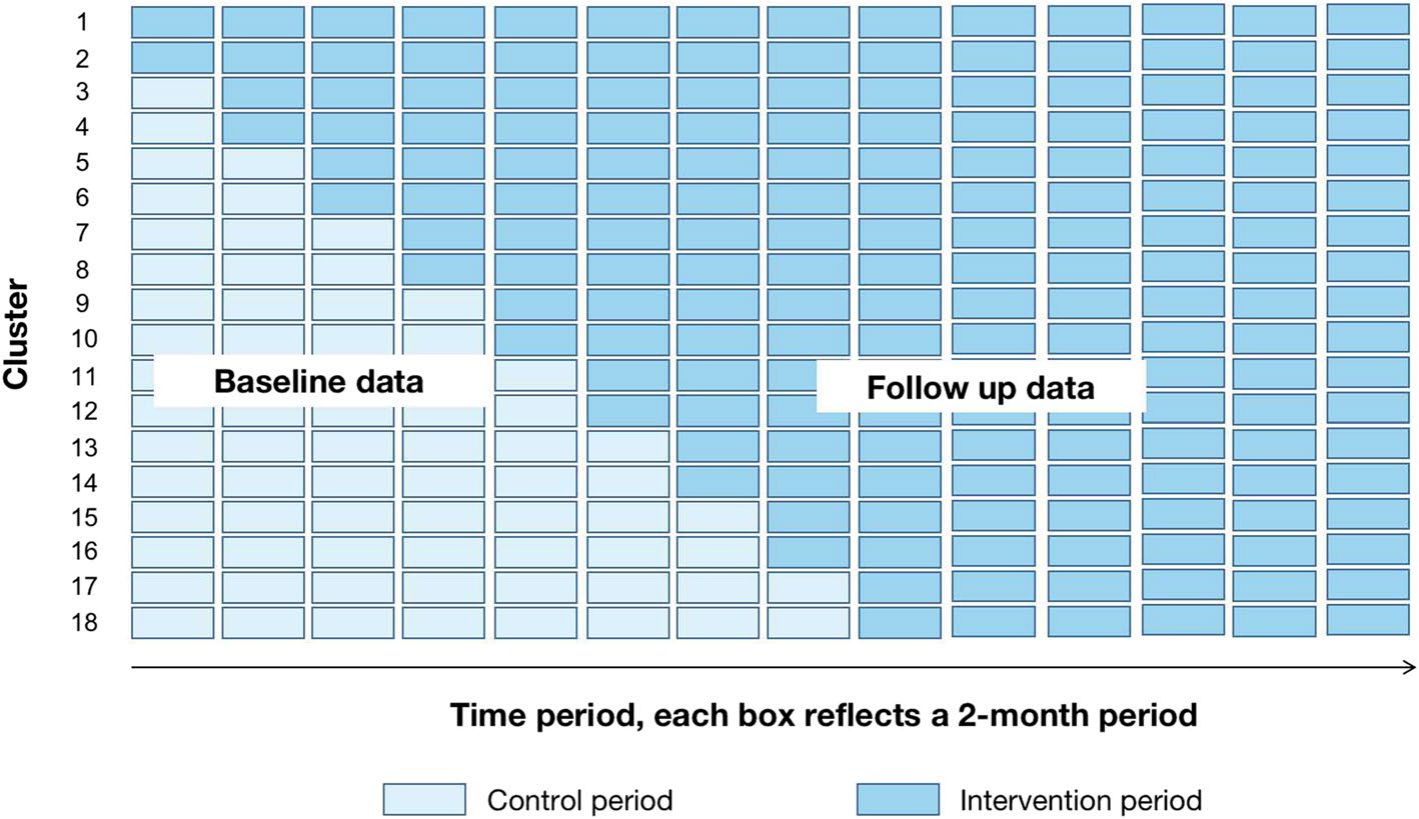
Preeclampsia risk prediction model for Chinese pregnant women (ChiPERM) stepped-wedge design

The study will be a randomized stepped-wedge controlled trial design conducted in eighteen hospitals in mainland China. The hospitals are geographically defined groupings of antenatal facilities with common obstetric management. As shown in Fig. 1, repeated cross-sectional outcomes data will be gathered on a monthly basis across all eighteen hospitals for the duration of the study (28 months). Baseline data will be collected for each hospital from prior to the commencement of the preeclampsia risk prediction in the first hospital to the start of the intervention in each sector. Stepped implementation of a 2-month practice change of preeclampsia risk prediction will be delivered in a randomly selected order at two monthly intervals. Follow-up data will continue to be collected for all eighteen hospitals following completion of the practice change intervention. The outcomes of the trial will be determined by comparing the incidence of preeclampsia outcomes between the baseline and follow up periods for the eighteen sectors combined.

A randomized stepped-wedge controlled trial design is recommended for the evaluation of clinical practice change interventions as it provides a number of pragmatic and scientific advantages over a randomized controlled trial design [17, 18]. First, it provides a similar level of evidence as a standard parallel cluster randomized controlled trial. Second, although all the participated hospitals will receive the intervention, its sequential implementation across eighteen sectors provides the capacity to identify secular trends, i.e. changes over time before the intervention is implemented. Third, the design provides an opportunity for all participating services and women to receive the intervention, overcoming ethical and logistical challenges arising from withholding the intervention.

All eighteen hospitals have covered 15 provinces across mainland China. The antenatal services in the eighteen hospitals service urban and rural areas and provide care to over 100,000 women annually, accounting for approximately 1% of births in mainland China.

### Random allocation and blinding

A statistician who is independent of intervention development and implementation will randomly allocate the order in which the Bayes-theorem based preeclampsia risk prediction is implemented across the eighteen hospitals. The random sequence will be generated using a computerized random number generator with allocation undertaken for all eighteen hospitals at the one time. Study personnel involved in collecting outcome data will be blind to the allocated order of the delivery of the preeclampsia risk prediction across the hospitals. Participants providing outcome data will be informed the experimental nature of preeclampsia risk prediction across hospitals and therefore will not be blind to the stage of study occurring in the service they attend. Given the practice change nature of the preeclampsia risk prediction, clinicians in antenatal services will be aware when their service is in the intervention period.

### Participant eligibility and recruitment

During the 28-month data collection period, in the hospitals conducting this risk prediction intervention, it is intended that all pregnant women with singleton pregnancy who attend prenatal visit before 13^+6^ gestational weeks will be inquire to participate this study. Those women signing the informed consent will receive the Bayes-theorem based preeclampsia risk prediction.

The inclusion criteria are: maternal age ≥ 18 years old, singleton pregnancy, and prenatal visit between 11–13^+6^ weeks of gestational age, and agreement to the informed consent. The exclusion criteria were: multiple pregnancy, first prenatal visit after 14 weeks of gestational age, with severe fetal abnormalities, regular taking aspirin before first antenatal visit, complicated with severe psychological diseases or other diseases which are inappropriate for the study. All enrolled women have the chance to decline participating in or opting out during followup period.

### Intervention

**Fig. 2:**
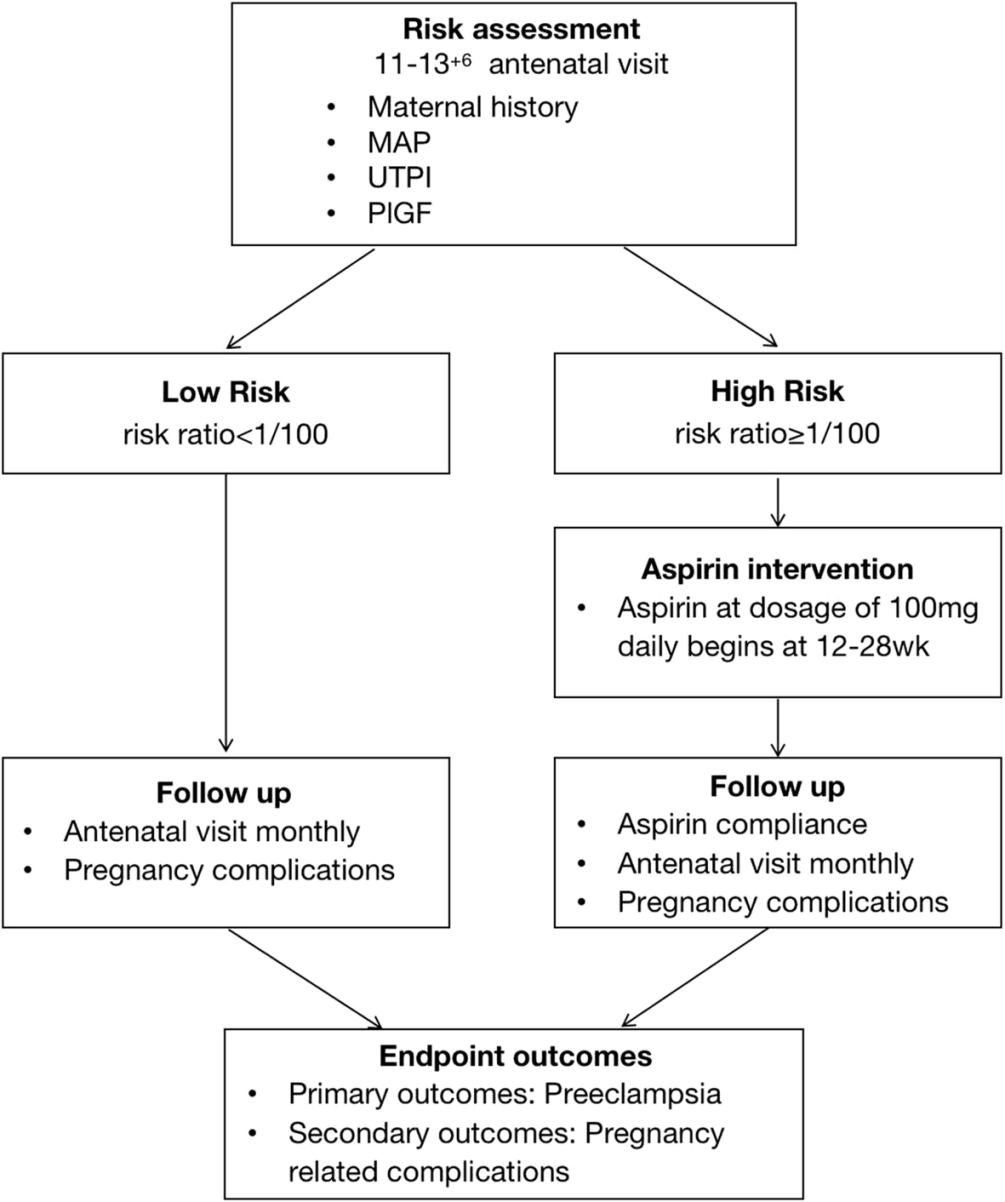
Bayes-theorem based preeclampsia risk preventive strategy

Model of care for addressing the Bayes-theorem based preeclampsia risk prediction in first trimester will be implemented in antenatal service during 11–13^+6^ gestational weeks across the eighteen participating sectors. As shown in Fig. 2, the prediction pipeline will consist of four key elements, including Bayes-theorem based risk assessment, aspirin intervention, followup and endpoint outcomes. The inventions will be delivered to women who attend antenatal visit each month and at delivery and till 42 day postpartum.

### Risk assessment

The implementation of the Bayes-theorem based preeclampsia risk prediction will be calculated by the model of The Fetal Maternal Foundation (https://fetalmedicine.org/research/assess/preeclampsia/first-trimester). This model is derived from maternal history, MAP, UTPI and serum PlGF at 11–13^+6^ weeks. Maternal history includes age, race, height, weight, gestational week based on crown rump length (CRL), pregnancy history, use of artificial reproductive technique, smoking history, history of chronic diseases (hypertension, diabetes mellitus, renal disease), adverse pregnancy history, previous history of preeclampsia, history of autoimmune diseases, pregnancy interval over 10 years, systolic blood pressure ≥ 130mmH and (or) diastolic blood pressure ≥ 80 mmHg, obstructive sleep apnea syndrome and previous history of prenatal diagnosis such as villus biopsy and amniocentesis.

The results of laboratory examination will be collected, including levels of folic acid, vitamin B12, fasting blood glucose, blood routine, urine routine, renal function, liver function, blood lipid and coagulation function. Blood pressure is measured by validated automated devices (OMRON, HBP-1320, China) according to a standardized protocol.

Uterine artery PI (UTPI) is measured by Doppler examination of uterine arteries technique in the period from 11 to 13^+6^ gestational weeks. Since this is a common time for first-trimester ultrasound examination in mainland China, UTPI measurement in this trial is practical and available. For the first-trimester transabdominal assessment of uterine artery resistance, a midsagittal section of the uterus and cervix is obtained initially. Using color flow mapping, the transducer is gently tilted sideways, in the purpose that the uterine arteries are identified with high-velocity blood flow along the side of the cervix and uterus. The pulsed-wave Doppler sampling gate should be narrow (set at approximately 2 mm) and positioned on either the ascending or descending branch of the uterine artery at the point closest to the internal cervical os, with an insonation angle < 30◦. In order to verify that the uterine artery is being examined, the peak systolic velocity should be > 60 cm/s. The PI is measured when at least three identical wave forms are obtained. Detailed methodology can be referred to the guideline and published papers [19, 20].

Serum PlGF (Aucheer C001) and sFlt-1 (Aucheer C002) were tested by immunoassay, respectively. Samples were collected by veni puncture into tubes containing ethylenediaminetetraacetic acid, centrifuged, and stored at -80℃. The most centrally located veni puncture sample within each of the 6 intervals of gestational age for each pregnant woman was used for analysis. Sample collection methods, biospecimen processing, and validation of the assays used were described in manufacturer’s handbooks.

### Aspirin intervention

Those women who assess as high risk (risk ratio ≥ 1/100) will be prescribed aspirin for preeclampsia prevention. The dosage is 100 mg daily begins at 12–16 gestation week. Those women of low risk (risk ratio < 1/100) will not been prescribed aspirin. All the recruited women will be followed up monthly.

### Followup

At monthly antenatal visit, those women will be asked to take their left aspirin pills to the hospital. The clinician will record their compliance through pill counting. All women, regardless of high risk or low risk, will be recorded of blood pressure and newly-onset complications. At 24–24^+6^, 30–36^+6^ gestational week, maternal serum will be stored for the measurement of PlGF and soluble fms-like tyrosine kinase 1 (sFlt-1).

### Endpoint outcomes

Endpoint outcomes are collected at delivery and till 42 days postpartum. Pregnancy outcomes will be recorded include the incidence of preeclampsia, eclampsia, HELLP syndrome (hemolysis, elevated liver enzymes and low platelet), preterm birth, fetal growth restriction/small for gestational age, postpartum hemorrhage, placental abruption, maternal heart failure, pulmonary edema, fetal demise, fetal death, neonatal death, neonatal asphyxia, neonatal intensive care unit (NICU).

### Control and contamination

#### Traditional preeclampsia prevention

Prior to implementation of the Bayes-theorem based preeclampsia risk prediction in each of the eighteen sectors, usual antenatal care for addressing maternal preeclampsia risk evaluation during pregnancy will be provided. Such care is likely to vary by antenatal service and clinician as several guidelines available are varied.

#### Potential for contamination

As PlGF measurement is not available in all eighteen sectors till the initiation of intervention, the Bayes-theorem based preeclampsia risk preventive strategy will not be accessible to clinicians during the baseline phase.

### Measures

#### Primary outcomes

The primary outcomes for this trial is the incidence of preeclampsia. The diagnosis will be collected during pregnancy period, at delivery and till 42 days postpartum, including the detailed onset time and severity. Related complications will also be recorded such as eclampsia and HELLP syndrome.

#### Secondary outcomes

There are obstetric, maternal, fetus/neonatal secondary outcomes for this trial, including the incidence of obstetric complications (preterm birth, placental abruption, postpartum hemorrhage), maternal complications (maternal heart failure, pulmonary edema), fetal complications (fetal growth restriction, small for gestational age, fetal demise, fetal death, neonatal death, neonatal asphyxia, NICU).

### Data collection procedures

#### Primary and secondary outcomes measures

Pregnancy outcomes will be recorded extracted from electronic medical records at delivery and postpartum outpatient visit. Experienced interviewers will confirm these information by telephone at 42 days in postpartum period. The postpartum survey will be pilot tested prior to starting the study to test comprehension, length and logic flow.

#### Process evaluation

At enrollment, women who attend their first antenatal visit before 13^+6^ weeks will be invited to participate in this study and sign an informed consent. Each weekday a sample of women who complete preeclampsia prediction test will be notified about their risk classification. Those women who are at high risk of preeclampsia predicted by the Bayes-theorem based risk model will be telephoned to book a visit for their obstetricians for aspirin prescription, while those women at low risk will be messaged about their results. In the follow-up period, all enrolled women will be contacted for a face-to-face interview for their obstetric complication and blood pressure measurement at their regular antenatal visit. Each weekday a sample of women who are at their 20–24^+5^ and 30–36^+6^ weeks for blood sample collection for PlGF and sFlt-1 measurement.

#### Data management

Data management protocol has been drafted and approved by advisory group of this trial. The whole process of data management will obey this protocol, and collected data will be stored according to the requirement of the Research Ethics Committee of Obstetrics and Gynecology Hospital of Fudan University. Data is accessible to principle investigator and statisticians.

#### Sample size

It is expected that 15,000 eligible pregnant women in all eighteen sectors will register at antenatal visit in first trimester (based on previous annual delivery number in each sector) during the first 18-month enrollment period of this trial. Assuming 70% of invited women will agree to participate in this project, approximately 10,500 women will be enrolled in this study.

### Statistical analysis

Statistical analyses will be conducted on an intention-to-treat basis. Primary and secondary outcomes will be analyzed using a logistic mixed model to determine the significance of the between group difference in the incidence of preeclampsia and other complications, with adjustment for the effect of the estimated risk of preeclampsia at screening and the participating center. Prespecified analyses will be performed in subgroups that were categorized according to the estimated risk of preterm preeclampsia and history of preeclampsia, and a post hoc subgroup analysis according to the participating centers. A sensitivity analysis will be performed to take into account the effect of withdrawal of consent and loss to follow-up. Kaplan–Meier estimates of the cumulative incidence of preeclampsia according to trial group will be conducted, in which deliveries that were not with preeclampsia were excluded. The statistical software package R was used for data analyses.

### Research trial governance

The advisory committee of this trial is consisted of obstetricians, statisticians, policy makers and clinical experts with expertise related to preeclampsia. The project team will implement strategies and collect data according to study protocol. Local clinical experts of all the eighteen participating sectors will provide advice and tailor the trail implementation strategies based on sector-specific requirements.

### Trial discontinuation or modification

This trial has been approved by the Research Ethics Committee of Obstetrics and Gynecology Hospital of Fudan University (2021–48). Each hospital of all the eighteen sectors will apply for ethic approval from their own hospital before the trial initiation. There are no criteria for trial discontinuation as it is not anticipated that any events would occur that would warrant discontinuing the trial. The researchers will notify all the unforeseen adverse events and timely report to the Research Ethics Committees of Obstetrics and Gynecology Hospital of Fudan University and of the local sector. The trial has been registered at Chinese Clinical Trial Registry (ChiCTR2100043520), and protocol modifications and deviations from original protocol will be updated.

## Discussion

Despite clinical guideline recommendations based on high-quality randomized controlled in Europe, effectiveness of the Bayes-theorem based preeclampsia risk prediction model in first trimester among Chinese women is limited. There is an urgent need for evaluate its effectiveness in reducing the risk of preeclampsia among Chinese pregnant women.

This will be the first randomized controlled trial to evaluate the effectiveness of the Bayes-theorem based risk model in China. The stepped-wedge design is logical, feasible and ethical in the setting of eighteen sectors providing maternal antenatal services. The key elements of trial are random allocation across the sectors, comprehensive data collection and management protocol.

If positive preventive effect in clinical practice are found, this evidence will support health service adoption of this risk prediction model to reduce the risk of preeclampsia among Chinese women.

## Data Availability

The datasets during the current study are available from the corresponding author on reasonable request.

## Abbreviations

APPEC: Aspirin in the Prevention of Pre-Eclampsia in China
FIGO: The International Federation of Gynecology and Obstetrics
MAP: Mean arterial pressure
UTPI: Uterine artery PI
PlGF: Serum placenta growth factor
FMF: Fetal Medicine Foundation
ChiPERM: Preeclampsia risk prediction model for Chinese pregnant women
CRL: Crown rump length
sFlt-1: Soluble fms-like tyrosine kinase 1
NICU: Neonatal intensive care unit
HELLP syndrome: Hemolysis, elevated liver enzymes and low platelet
